# Long-Term Outcome of Epiretinal Membrane Surgery in Patients with Internal Limiting Membrane Dehiscence

**DOI:** 10.3390/jcm9082470

**Published:** 2020-08-01

**Authors:** Min-Woo Lee, Il Jung, Yong-Yeon Song, Seung-Kook Baek, Young-Hoon Lee

**Affiliations:** Department of Ophthalmology, Konyang University College of Medicine, Daejeon 35365, Korea; bogus1105@gmail.com (M.-W.L.); jungil@kyuh.ac.kr (I.J.); legend-syy@hanmail.net (Y.-Y.S.); backkka@hanmail.net (S.-K.B.)

**Keywords:** ERM, ILM dehiscence, RNFL schisis

## Abstract

Purpose: To identify the effect of internal limiting membrane (ILM) dehiscence on surgical outcomes in eyes that have undergone epiretinal membrane (ERM) removal. Methods: Consecutive eyes with performed vitrectomy for ERM removal were included. Subjects were divided into two groups: patients with ILM dehiscence (group 1) and without ILM dehiscence (group 2). The best-corrected visual acuity (BCVA) and retinal layer thickness before and after surgery were compared between the two groups. Results: A total of 86 eyes were enrolled. Forty-six eyes (53.5%) showed ILM dehiscence before surgery. The baseline BCVAs were 0.46 ± 0.29 and 0.45 ± 0.25 in groups 1 and 2, respectively (*p* = 0.801). The BCVAs at 3, 6, and 12 months after surgery differed significantly between the two groups. The subfoveal thickness and inner retinal layer thickness (IRLT) of group 1 vs. 2 were 507.4 ± 80.0 vs. 417.6 ± 63.6 μm, and 270.2 ± 74.3 vs. 182.6 ± 60.4 μm, respectively (both *p* < 0.001). These differences between the groups remained significant until 12 months after surgery. In multivariate analyses, the location of ILM dehiscence (B = −0.105, *p* = 0.034) and final IRLT (B = 0.001, *p* = 0.046) were significant factors affecting the final BCVA. Conclusions: ILM dehiscence is a relatively common finding and associated with preoperative and postoperative increased IRLT, which results in worse surgical outcomes compared to the absence of ILM dehiscence in patients with ERM. Additionally, the final BCVA was significantly affected by its location and final IRLT in patients with ILM dehiscence.

## 1. Introduction

Idiopathic epiretinal membrane (ERM) is a common macular disorder, occurring in approximately 7% of the population. [1] It is characterized by fibrocellular proliferation on the inner retinal surface of the macular area, which may induce contraction of the retina. [1,2] Such contractions of the ERM can induce significant tangential traction on the internal limiting membrane (ILM) and blood vessels, causing visual disturbances and metamorphopsia [3,4]. Surgical removal of the ERM is a well-known standard procedure for improving symptoms by releasing the associated tractional force. However, functional outcomes are sometimes unsatisfactory, despite ERM removal with anatomical success. 

Therefore, several prognostic factors, such as preoperative visual acuity, symptom duration, central foveal thickness, disruption of the photoreceptor inner/outer segment (IS/OS) junction or cone outer segment tips (COST) line, ectopic inner foveal layer (EIFL), and inner retinal layer thickness (IRLT), have been reported to affect visual outcomes [5,6,7,8,9,10,11,12]. Shimozono et al. [7] reported that the status of the COST line was strongly correlated with visual acuity after ERM surgery, indicating its usefulness as a prognostic factor together with the IS/OS junction. Yang et al. [9] found that postoperative visual outcomes were well correlated with the preoperative central IRLT and postoperative restoration of the inner retinal layer configuration after ERM peeling. Govetto et al. [11] reported that the presence of EIFL should be considered a negative prognostic factor for ERM surgery. Recently, Hussnain et al. [13] reported that the schisis of the retinal nerve fiber layer (RNFL) is a relatively common occurrence in ERM and frequently associated with areas of dehisced ILM. However, few studies have addressed the effects of preoperative dehisced ILM, or ILM tears, on visual and anatomical outcomes after ERM removal.

The purpose of this study was to identify the effect of preoperative ILM dehiscence on long-term visual and anatomical outcomes in eyes with performed ERM removal by comparing the visual acuity and retinal layers of patients with ILM dehiscence and patients without ILM dehiscence.

## 2. Methods

### 2.1. Patients

This retrospective observational study adhered to the tenets of the Declaration of Helsinki and was approved by the Institutional Review Board of Konyang University Hospital, Daejeon, Korea. We reviewed the charts of patients with consecutive ERM treated with 25-gauge pars plana vitrectomy by a single surgeon (Y.-H.L.) at Konyang University Hospital from January 2015 to December 2018. Among these patients, we enrolled those who were diagnosed with idiopathic ERM and followed up for at least 12 months postoperatively. We recorded detailed histories and best-corrected visual acuity (BCVA), intraocular pressure, spherical equivalent, and axial length. All patients underwent 25-gauge vitrectomy and complete ERM removal, along with ILM peeling using indocyanine green. ILM peeling was performed from the intact ILM to the area of ILM dehiscence, and initiation of peeling from the area of ILM dehiscence was avoided. When combined with cataract surgery, phacoemulsification was performed before vitrectomy, and the combined surgery was performed in patients with grade 2 or higher lens opacity classification [LOC III]. Subjects were divided into two groups: patients with ILM dehiscence (group 1) and patients without ILM dehiscence (group 2). The stages of ERM were classified according to the EIFL as in previous studies as follows: Stage 1, mild ERM with preserved foveal depression; Stage 2, loss of foveal depression, but all retinal layers are well defined; Stage 3, continuous EIFL over whole foveal floor; Stage 4, advanced ERM with complete foveal disorganization [10,11]. Exclusion criteria were a history of ophthalmic disease other than ERM, such as retinal detachment, inflammatory eye disorder, myopic schisis, vitreomacular traction syndrome, full-thickness macular hole, retinal vessel occlusion, glaucoma, severe cataract potentially affecting BCVA, and previous intraocular surgery excluding cataract extraction. We also excluded patients with high myopia with an axial length ≥ 26.0 mm or BCVA ≤ 20/200 before surgery.

### 2.2. Analyses of ILM Dehiscence and Optical Coherence Tomography (OCT) Measurements

**Figure 1:**
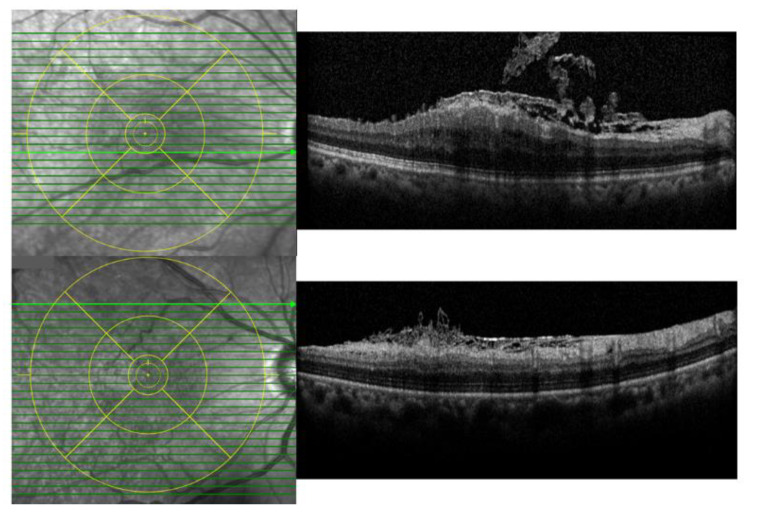
Representative images of internal limiting membrane (ILM) dehiscence with retinal nerve fiber layer schisis. The upper row shows ILM dehiscence within the inner circle of Early Treatment of Diabetic Retinopathy Study (ETDRS) and the lower row shows ILM dehiscence outside the inner circle of ETDRS.

Two independent observers (M.-W.L. and Y.-H.L.) performed detailed analyses using multimodal imaging of these cases retrospectively. ILM dehiscence was diagnosed using spectral-domain OCT by observing hyporeflective linear spaces of varying lengths and depths interspersed with hyperreflective bands, and a protrusion of RNFL tissue projecting into the posterior vitreous cavity (spaghetti sign), as described in a previous study (Figure 1) [13].

B-scan images were examined and correlated with color fundus photography and en face imaging findings. The size of the ILM dehiscence was considered focal if it was within a disc diameter on spectral-domain OCT, or diffuse if it exceeded it. We also investigated the location of ILM dehiscence as to whether it existed in the inner circle of Early Treatment of Diabetic Retinopathy Study (ETDRS) or not. 

Spectral-domain OCT (Spectralis; Heidelberg Engineering, Heidelberg, Germany) was performed before surgery and at 1, 3, 6, and 12 months after surgery, using the AutoRescan mode. The AutoRescan feature of the Spectralis OCT device provides an OCT scan and corresponding high-quality fundus picture and relies on active eye tracking. The images were generated using the horizontal OCT cross-section (25 lines spaced 240 μm apart). The thickness of the segmented subfoveal retinal layer, the central circle of ETDRS, was measured automatically using the HRA/Spectralis Viewing Module (ver. 6.9.5.0). The manual adjustment was performed when obvious segmentation error was found. The inner retinal layer was defined as the RNFL + ganglion cell layer (GCL) + inner plexiform layer (IPL) + inner nuclear layer (INL). Images with any motion artifact, involuntary saccade, obvious decentration misalignment, or algorithm segmentation failure considered inappropriate to analyze despite manual adjustment on B-scan images were excluded.

The continuity of the IS/OS junction and COST line were evaluated on preoperative SD-OCT images for the diagnosis of photoreceptor layer (PRL) disruption. A disruption of the line was indicated when there was a loss of individual hyperreflective lines in the fovea as described in a previous study [7]. 

### 2.3. Statistical Analyses

Demographic characteristics and ocular parameters were compared using the independent *t*-test and chi-squared test. Pearson’s correlation analyses were performed to identify the relationships between each retinal layer and the final BCVA. Univariate and multivariate linear regression analyses were performed to identify factors associated with final BCVA in patients with ILM dehiscence. Univariate and multivariate logistic regression analyses were also performed to determine the factors associated with ILM dehiscence. All statistical analyses were performed using SPSS software (version 18.0; IBM Corp., Armonk, NY, USA).

## 3. Results

### 3.1. Demographics

A total of 86 eyes were enrolled, of which 46 (53.5%) showed ILM dehiscence before surgery (Table 1).

The mean ages in group 1 and 2 were 65.3 ± 9.3 and 67.9 ± 9.1 years, respectively, and did not differ significantly (*p* = 0.059). Sex, laterality, spherical equivalent, intraocular pressure, and axial length were not also significantly different between the two groups. In group 1, Stage 1 ERM were classified in 2 eyes (4.3%), Stage 2 ERM in 12 eyes (26.1%), Stage 3 ERM in 20 eyes (43.5%), and Stage 4 ERM in 12 eyes (26.1%). In group 2, Stage 1 ERM were classified in 5 eyes (12.5%), Stage 2 ERM in 9 eyes (22.5%), Stage 3 ERM in 22 eyes (55.0%), and Stage 4 ERM in 4 eyes (10.0%); the distribution of ERM stages was not significantly different between the two groups (*p* = 0.116). Twenty-eight eyes (60.9%) in group 1 and 28 eyes (70.0%) in group 2 showed mild cataracts (1.7 ± 0.5 vs. 1.8 ± 0.5 using the lens opacity classification [LOC III], *p* = 0.631), and they underwent vitrectomy combined with phacoemulsification. Group 1 showed significantly thicker baseline subfoveal thickness (SFT) and IRLT than those in group 2. PRL disruption was observed in 24 eyes (52.2%) of group 1 and 17 eyes (42.5%) of group 2. In group 1, 26 eyes (56.5%) showed a diffuse ILM dehiscence, which was larger than one disc diameter, and 22 eyes (47.8%) had an ILM dehiscence within the inner circle of ETDRS.

### 3.2. Changes in Visual Acuity, SFT, and IRLT after Surgery

Baseline BCVAs were 0.46 ± 0.29 and 0.45 ± 0.25 in group 1 and 2, respectively (*p* = 0.801) (Table 2), and those at 1 month after surgery were 0.24 ± 0.21 and 0.18 ± 0.16, respectively (*p* = 0.137). 

**Figure 2:**
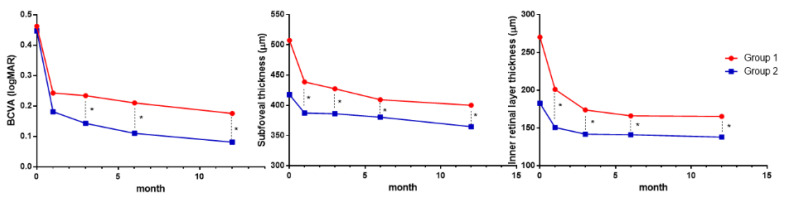
Best-corrected visual acuity (BCVA), subfoveal thickness, and inner retinal layer thickness at baseline (preoperative), 1, 3, 6, and 12 months postoperatively in epiretinal membrane patients with ILM dehiscence (group 1) and patients without ILM dehiscence (group 2). * *p* < 0.05.

At 3 months, the BCVAs were 0.23 ± 0.21 and 0.14 ± 0.14 in groups 1 and 2, respectively, and differed significantly (*p* = 0.019) (Figure 2).

The BCVA at 6 and 12 months after surgery were also significantly different between the two groups.

**Figure 3:**
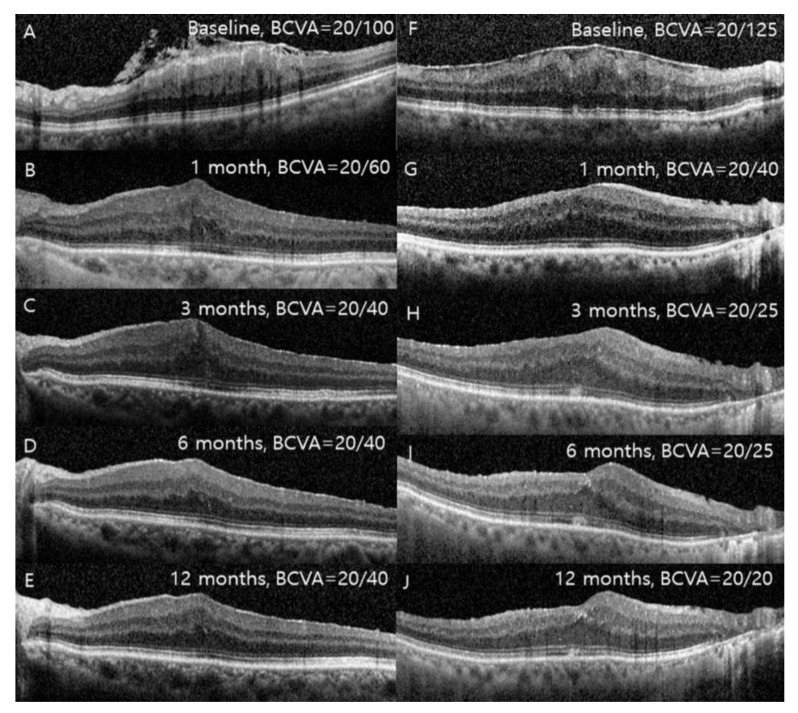
Consecutive optical coherence tomography images in epiretinal membrane patients with internal limiting membrane (ILM) dehiscence (**A**–**E**) and without ILM dehiscence (**F**–**J**). In the patients with ILM dehiscence, a relatively thick inner retinal layer thickness was maintained compared to the patients without ILM dehiscence.

The SFTs were 507.4 ± 80.0 and 417.6 ± 63.6 μm in groups 1 and 2, and the IRLTs were 270.2 ± 74.3 and 182.6 ± 60.4 μm, respectively (both *p* < 0.001). These differences between the groups remained throughout the follow-up period (at 1, 3, 6, and 12 months). The changes in the BCVA, SFT, and IRLT from baseline to 12 months after surgery were significantly different between the groups: 0.29 vs. 0.37 (*p* = 0.044), 107.3 vs. 52.9 (*p* < 0.001), and 104.9 vs. 44.5 (*p* < 0.001), respectively (Figure 3).

### 3.3. Thickness of Each Retinal Layer at 12 Months after Surgery

The SFT and IRLT in group 1 and 2 were 400.2 ± 47.1 and 364.8 ± 53.7 μm (*p* = 0.002) and 165.4 ± 41.2 and 138.1 ± 44.2 μm (*p* = 0.004), respectively (Table 3). 

In groups 1 and 2, the thicknesses of the RNFL were 28.8 ± 23.6 and 22.7 ± 19.1 μm (*p* = 0.189), those of GCL were 43.5 ± 11.5 and 35.9 ± 15.2 μm (*p* = 0.010), those of IPL were 40.3 ± 10.1 and 35.6 ± 10.2 μm (*p* = 0.003), and those of INL were 52.9 ±12.0 and 46.0 ± 13.9 μm (*p* = 0.016), respectively; these differences were significant with the exception of RNFL thickness. The thicknesses of the outer plexiform layer (OPL), outer nuclear layer (ONL), PRL, and retinal pigment epithelium layer (RPE) were not significantly different between the two groups.

**Figure 4:**
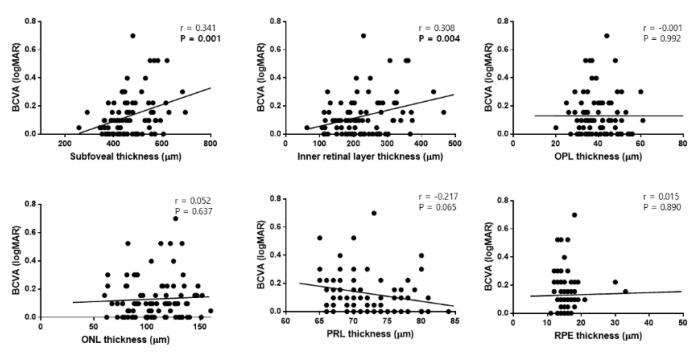
Scatter plots showing the associations between preoperative retinal layer thicknesses and the final best-corrected visual acuity (BCVA) in patients with epiretinal membrane. Preoperative subfoveal thickness and inner retinal layer thickness were significantly correlated with the final BCVA. OPL, outer plexiform layer; ONL, outer nuclear layer; PRL, photoreceptor layer; RPE, retinal pigment epithelium.

In Pearson’s correlation analyses, the baseline SFT (r = 0.341, *p* = 0.001), baseline IRLT (r = 0.308, *p* = 0.004), and PRL disruption before surgery (r = 0.236, *p* = 0.029) were significantly correlated with the final BCVA (Figure 4).

**Figure 5:**
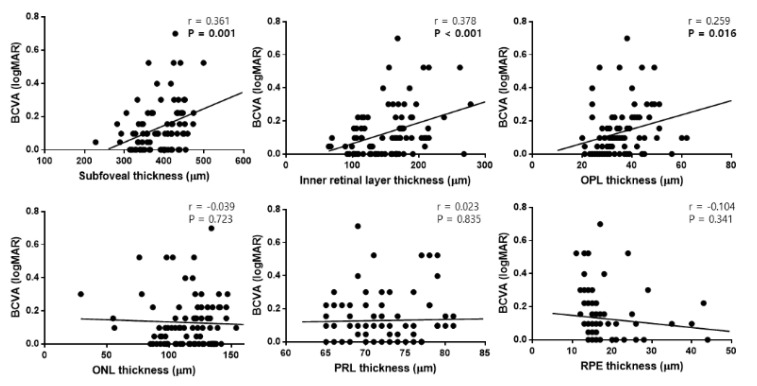
Scatter plots showing the associations between retinal layer thicknesses at 12 months postoperatively and the final best-corrected visual acuity (BCVA) in patients with epiretinal membrane. Subfoveal thickness, inner retinal layer thickness, and outer plexiform layer (OPL) thickness were significantly correlated with the final BCVA. ONL, outer nuclear layer; PRL, photoreceptor layer; RPE, retinal pigment epithelium.

Additionally, SFT (r = 0.361, *p* = 0.001), IRLT (r = 0.378, *p* < 0.001), and OPL (r = 0.259, *p* = 0.016) thicknesses at 12 months after surgery were correlated with the final BCVA (Figure 5).

### 3.4. Univariate and Multivariate Linear Regression Analyses in Patients with ILM Dehiscence

In univariate analyses, baseline BCVA (B = 0.174, *p* = 0.045), baseline SFT (B = 0.001, *p* = 0.045), baseline IRLT (B = 0.001, *p* = 0.036), location of the ILM tear (B = −0.135, *p* = 0.007), photoreceptor layer disruption (B = 0.101, *p* = 0.045), final SFT (B = 0.001, *p* = 0.020), and final IRLT (B = 0.002, *p* = 0.009) were significant factors associated with the final BCVA (Table 4).

These factors showed variance inflation factors ≤ 3, which indicates low collinearity. In multivariate analyses, the location of ILM dehiscence (B = −0.105, *p* = 0.034) and the final IRLT (B = 0.001, *p* = 0.046) were significant factors affecting the final BCVA.

### 3.5. Univariate and Multivariate Logistic Regression Analyses for Determining the Factors Associated with ILM Dehiscence

In univariate analyses, age (OR = 0.947, *p* = 0.027), baseline SFT (OR = 1.020, *p* < 0.001), baseline IRLT (OR = 1.021, *p* < 0.001), final BCVA (OR = 55.190, *p* = 0.004), final SFT (OR = 0.015, *p* = 0.002), and final IRLT (OR = 1.018, *p* = 0.003) were significant factors associated with ILM dehiscence (Table 5).

In multivariate analyses, baseline IRLT (OR = 1.018, *p* < 0.001) and final BCVA (OR = 83.987, *p* = 0.039) showed a significant result.

## 4. Discussion

ERM is a relatively common disease and has been investigated and treated actively in retina clinics worldwide, including our hospital. We found that there are many patients with ERM showing ILM dehiscence and protrusion of RNFL tissue; however, few ILM dehiscence studies or related issues have been reported to date. Thus, we attempted to identify the characteristics and impact of ILM dehiscence on surgical outcomes. Our findings indicated that it was relatively common (53.5%) and was related to a delay in postoperative restoration of the inner retinal layer, which may result in a low visual outcome.

Regarding the mechanism of the ILM dehiscence, Bovey et al. [14] hypothesized that the partial vitreous detachment induces detachment of the ILM by exerting traction on the ILM; partial separation then occurs between the ILM and ERM, which remains adherent to the posterior hyaloid. The ILM becomes folded on itself due to the absence of supportive tissue. However, further studies are needed to clarify the exact mechanism of occurrence of ILM dehiscence and to prove the relationship between vitreous detachment and ILM dehiscence. Recently, Hussnain et al. [13] reported schisis of the RNFL in 12 of 21 eyes (57.1%) in their consecutive ERM series, which was observed intraoperatively as areas of negative staining with brilliant blue G. ILM dehiscence, related to schisis or protrusion of the RNFL, is a relatively common finding in ERM patients; thus, surgeons should be cautious preoperatively and intraoperatively, as this condition can affect the surgical outcome. 

The thicknesses of the outer retinal layers, including the OPL, ONL, PRL, and RPE, were not significantly different at baseline or 12 months after surgery between the two groups. However, SFT and IRLT showed significant differences at baseline, and these differences remained until 12 months postoperatively. The previous study reported that identifying the spaghetti sign on SD-OCT before surgery should alert the surgeon that there is ILM rupture, and therefore ILM peeling should not be performed directly over these areas to prevent damage to the underlying exposed neurosensory retina [13].

Meanwhile, although the surgeon (Y.-H.L.) avoided initiation of peeling at this area, and signs of ILM dehiscence such as protrusion of the RNFL had disappeared after surgery, the IRLT was consistently thicker in group 1 until 12 months after surgery; this may be an indication of delayed recovery or irreversible damage to the inner retina. The inner retinal layer can become damaged by ILM dehiscence itself or the process by which ILM dehiscence occurs before surgery, and such a damaged inner retina would be more vulnerable to vertical force during ERM or ILM peeling. In patients with ERM showing ILM dehiscence, more severe damage to the inner retina may be inevitable before and during surgery, compared with patients without ILM dehiscence.

The BCVAs at baseline and 1 month after surgery were not significantly different between the two groups. However, there was a consistent difference in the BCVA at three months after surgery between the groups, and final BCVA was a significant factor associated with ILM dehiscence in multivariate logistic regression analyses. Additionally, the final BCVA was significantly correlated with both the baseline and final IRLT. Joe et al. [15] explained the relationship between visual acuity and IRLT as follows: when the tangential traction becomes intense, the inward peak of the ONL becomes exaggerated, resulting in the attachment of adjacent parafoveal inner retinal layers, then the cells in each retinal layer lose polarity and normal neural transmission fails. Recovery of the polarity and neural transmission by removal of traction may be insufficient in patients with ILM dehiscence because of delayed restoration of the inner retinal layer compared with those without ILM dehiscence. Although further studies are needed to prove it, delayed recovery or permanent damage to the inner retina in patients with ILM dehiscence may contribute to a poor visual prognosis.

The preoperative and postoperative thicknesses of the outer retinal layers were not significantly correlated with the final BCVA. However, preoperative PRL disruption, including IS/OS junction and COST line, was significantly correlated with the final BCVA. Kim et al. [8] reported that P1 implicit time in ERM patients with IS/OS disruption was significantly delayed, which suggests the development of Muller/bipolar cell abnormalities causing neural transmission delay and poor visual recovery. Shimozono et al. [7] reported that the status of the COST line was strongly correlated with the BCVA after ERM surgery, indicating its usefulness as a prognostic factor in conjunction with the IS/OS junction. In this study, the final BCVA of patients with ILM dehiscence was also significantly affected by PRL disruption in univariate analyses, and the number of cases with PRL disruption was not significantly different between the two groups. ILM dehiscence and PRL disruption seemed to have less of a direct relationship; however, PRL disruption would be a useful prognostic factor in patients with ERM, regardless of ILM dehiscence. 

Twenty-six cases (56.5%) showed diffuse ILM dehiscence, and the ILM dehiscence involved the inner circle of ETDRS in 22 cases (47.8%) in group 1. Whereas the size of ILM dehiscence was not significantly associated with the final BCVA, its location significantly affected the final BCVA in the multivariate analyses, suggesting worse visual prognosis when ILM dehiscence involves the inner circle of ETDRS. Hussnain et al. [13] reported that 12 of 21 cases (57.1%) of RNFL schisis involved the papillomacular bundle, defined as the area between lines drawn horizontally toward the macula from the upper and lower borders of the optic disc. However, they did not analyze its relationship with visual prognosis. If the location is close to the macular center, the recovery of the foveola after surgery could be affected, resulting in a bad prognosis for central vision. The preoperative location of ILM dehiscence, whether it involves the inner circle of ETDRS or not, is quite intuitive and can be observed easily in a relatively short time using en face and B-scan images. Therefore, the presence of ILM dehiscence and its location could be useful factors for predicting visual outcomes after surgery in patients with ERM.

The final BCVA was also significantly affected by the final IRLT in multivariate analyses in patients with ILM dehiscence. Kim et al. [16] reported that the presence of fibrillary changes alternating hyperreflective and hyporeflective signals between ERM and retinal surface not parallel to the retinal surface, corresponding to ILM dehiscence in our study, resulted in a 25.5-fold increase in surgical removal difficulty compared with its absence. Therefore, surgeons should be cautious when peeling ERM or ILM in patients with ILM dehiscence to minimize inner retinal damage. The previous study reported that by carefully grasping the band of the ILM and drawing it horizontally towards the ERM zone, the membrane and ILM could be completely and easily removed from the macular zone. [14] However, such ILM band would contain some RNFL tissue, and the area around the ILM dehiscence may be RNFL schisis, which could induce more severe inner retinal damage during surgery. Another study reported that an attempt to start a flap or peel over these areas with forceps or membrane scrapers could potentially cause direct trauma to the RNFL, resulting in additional RNFL injury and scotoma. [13] Therefore, minimizing inner retinal damage by avoiding initiation of peeling the area near the ILM dehiscence during surgery would be helpful for a better visual prognosis.

Recent studies reported the EIFL as a bad prognostic factor for ERM surgery [10,11,12,17]. Oguizi et al. reported that the spectral-domain OCT staging system according to the presence of EIFL is effective for grading retinal damage and visual loss in eyes with ERM. [17] Gonzalez et al. [12] also reported that the EIFL staging scheme is an easy, fast, and reproducible method to evaluate visual prognosis with ERM surgery. The ERM stage according to the EIFL was also significantly associated with final BCVA in total patients in our study (B = 0.042, *p* = 0.024; this is not shown in the result section.) However, the ERM stage was not a significant factor associated with final BCVA in patients with ILM dehiscence, and was not also a significant factor associated with ILM dehiscence. Although the ERM stage is a useful factor for the prognosis of ERM surgery, the location of ILM dehiscence and final IRLT may be more significant factors associated with final BCVA in patients with ILM dehiscence.

Our study had several limitations. First, the retrospective nature of the work inevitably introduced some selection bias. Second, we could not evaluate various visual symptoms induced by the ERM such as metamorphopsia or scotoma. Third, there may be some fine autosegmentation errors in analyzing retinal thickness, although we performed manual adjustment and excluded images with a definite segmentation error. Fourth, although ERM stages were not significantly different between the two groups, group 1 showed a more advanced stage, which could be a confounding factor. The strength of our study is that we investigated long-term surgical outcomes via regular follow-ups and identified factors affecting the final BCVA in patients with ILM dehiscence, which has not been reported as far as we know. 

In conclusion, ILM dehiscence is a relatively common finding and is associated with preoperative and postoperative increased IRLT, which results in worse surgical outcomes compared with the absence of ILM dehiscence in patients with ERM. Additionally, the final BCVA was significantly affected by the location of ILM dehiscence and final IRLT in patients with ILM dehiscence. Therefore, in patients with ERM, surgeons should consider the presence and location of ILM dehiscence before surgery and minimize damage to the inner retinal layer by avoiding this area at initiation of ILM peeling for better visual outcomes.

## Figures and Tables

**Table 1:** Baseline characteristics and demographics.

	Group 1 (*n* = 46)	Group 2 (*n* = 40)	*p*-Value
Age	65.5 ± 9.3	67.9 ± 9.1	0.059
Sex (men, %)	17 (37.0%)	21 (52.5%)	0.076
Hypertension (n, %)	17 (37.0%)	18 (45.0%)	0.513
Diabetes (n, %)	6 (13.0%)	8 (20%)	0.400
Laterality (right, %)	26 (56.5%)	21 (52.5%)	0.709
Spherical equivalent	−0.34 ± 1.59	−0.14 ± 1.69	0.570
BCVA	0.46 ± 0.29	0.45 ± 0.25	0.801
Intraocular pressure	14.7 ± 3.0	13.8 ± 3.6	0.210
Axial length	23.4 ± 1.1	23.5 ± 0.9	0.704
Lens status (phakic, %)	28 (60.9%)	28 (70.0%)	0.497
Photoreceptor layer disruption (%)	24 (52.2%)	17 (42.5%)	0.395
Stage of ERM	2.9 ± 0.8	2.6 ± 0.8	0.116
Retinal layer thickness			
SFT	507.4 ± 80.0	417.6 ± 63.6	**<0.001**
IRLT	270.2 ± 74.3	182.6 ± 60.4	**<0.001**
OPL	40.2 ± 7.1	38.9 ± 9.0	0.449
ONL	110.4 ± 24.5	104.6 ± 24.2	0.274
PRL	72.0 ± 4.8	72.1 ± 4.2	0.995
RPE	15.6 ± 2.8	15.8 ± 3.3	0.778

Group 1, patients with an internal limiting membrane tear; group 2, patients without internal limiting membrane tear. BCVA, best-corrected visual acuity; ERM, epiretinal membrane; SFT, subfoveal thickness; IRLT, inner retinal layer thickness; OPL, outer plexiform layer; ONL, outer nuclear layer; PRL, photoreceptor layer; RPE, retinal pigment epithelium. Values in boldface (*p* < 0.050) are statistically significant.

**Table 2:** Changes in visual acuity, subfoveal thickness, and inner retinal layer thickness after surgery.

	Group 1			Group 2					
	BCVA	SFT	IRLT	BCVA	SFT	IRLT	*p*-Value *	*p*-Value ^†^	*p*-Value ^‡^
Baseline	0.46 ± 0.29	507.4 ± 80.0	270.2 ± 74.3	0.45 ± 0.25	417.6 ± 63.6	182.6 ± 60.4	0.801	**<0.001**	**<0.001**
1 month	0.24 ± 0.21	438.8 ± 62.9	201.1 ± 54.9	0.18 ± 0.16	387.3 ± 71.8	150.7 ± 46.9	0.137	**0.001**	**<0.001**
3 months	0.23 ± 0.21	427.5 ± 56.2	173.9 ± 42.1	0.14 ± 0.14	386.1 ± 69.7	141.9 ± 43.2	**0.019**	**0.003**	**0.001**
6 months	0.21 ± 0.22	409.4 ± 53.2	166.2 ± 40.9	0.11 ± 0.12	380.5 ± 60.7	141.1 ± 44.0	**0.014**	**0.021**	**0.010**
12 months	0.18 ± 0.17	400.2 ± 47.1	165.4 ± 41.2	0.08 ± 0.09	364.8 ± 53.7	138.1 ± 44.2	**0.002**	**0.002**	**0.004**

Group 1, patients with an internal limiting membrane tear; group 2, patients without an internal limiting membrane tear. BCVA, best-corrected visual acuity; SFT, subfoveal thickness; IRLT, inner retinal layer thickness. * *p*-values for BCVA differences; ^†^ *p*-values for SFT differences; ^‡^ *p*-values for IRLT differences. Values in boldface (*p* < 0.050) are statistically significant.

**Table 3:** Retinal layer thickness in each group at 12 months after surgery.

	Group 1	Group 2	*p*-Value
SFT	400.2 ± 47.1	364.8 ± 53.7	**0.002**
IRLT	165.4 ± 41.2	138.1 ± 44.2	**0.004**
RNFL	28.8 ± 23.6	22.7 ± 19.1	0.189
GCL	43.5 ± 11.5	35.9 ± 15.2	**0.010**
IPL	40.3 ± 10.1	35.6 ± 10.2	**0.003**
INL	52.9 ± 12.0	46.0 ± 13.9	**0.016**
OPL	36.7 ± 8.8	33.2 ± 8.8	0.070
ONL	114.9 ± 22.6	113.7 ± 21.3	0.807
PRL	73.0 ± 4.3	72.3 ± 4.3	0.498
RPE	17.6 ± 6.5	17.2 ± 6.2	0.727

Group 1, patients with an internal limiting membrane tear; group 2, patients without an internal limiting membrane tear. SFT, subfoveal thickness; IRLT, inner retinal layer thickness; RNFL, retinal nerve fiber layer; GCL, ganglion cell layer; IPL, inner plexiform layer; INL, inner nuclear layer; OPL, outer plexiform layer; ONL, outer nuclear layer; PRL, photoreceptor layer; RPE, retinal pigment epithelium. Values in boldface (*p* < 0.050) are statistically significant.

**Table 4:** Univariate and multivariate linear regression analyses in patients with an internal limiting membrane dehiscence.

	Univariate		Multivariate	
	B (95% CI)	*p*-Value	B (95% CI)	*p*-Value
Age	0.002 (−0.004–0.008)	0.457		
Sex	0.054 (−0.060–0.168)	0.344		
Hypertension	0.018 (−0.089–0.125)	0.738		
Diabetes	−0.037 (−0.190–0.116)	0.628		
Laterality	−0.015 (−0.120–0.089)	0.767		
SE	−0.017 (−0.050–0.015)	0.286		
IOP	−0.001 (−0.018–0.017)	0.922		
Axial length	0.003 (−0.045–0.050)	0.902		
Baseline BCVA	0.174 (0.004–0.344)	**0.045**	0.109 (−0.082–0.300)	0.254
Baseline SFT	0.001 (<0.001–0.001)	**0.027**	<0.001 (−0.002–0.001)	0.353
Baseline IRLT	0.001 (<0.001–0.001)	**0.036**	−0.001 (−0.002–0.000)	0.555
Lens status	0.063 (−0.0420–0.167)	0.231		
Tear size	0.073 (−0.029–0.175)	0.159		
Tear location	−0.135 (−0.231−0.039)	**0.007**	−0.105 (−0.203−0.008)	**0.034**
PRL disruption	0.101 (0.002–0.200)	**0.045**	0.069 (−0.024–0.162)	0.144
Stage of ERM	0.051 (−0.009–0.112)	0.094		
Final SFT	0.001 (< 0.001–0.002)	**0.020**	<0.001 (−0.002–0.002)	0.725
Final IRLT	0.002 (0.001–0.003)	**0.009**	0.001 (0.000–0.002)	**0.046**

CI, confidence interval; SE, spherical equivalent; IOP, intraocular pressure; BCVA, best-corrected visual acuity; SFT, subfoveal thickness; IRLT, inner retinal layer thickness; PRL, photoreceptor layer; ERM, epiretinal membrane. Values in boldface (*p* < 0.050) are statistically significant.

**Table 5:** Univariate and multivariate logistic regression analyses for determining the factors associated with internal limiting membrane dehiscence.

	Univariate		Multivariate	
	B (OR)	*p*-Value	B (95% CI)	*p*-Value
Age	−0.055 (0.947)	0.027	−0.051 (0.950)	0.103
Sex	1.158 (3.184)	0.100		
Hypertension	0.448 (1.565)	0.310		
Diabetes	0.455 (1.576)	0.441		
Laterality	0.265 (1.303)	0.542		
SE	−0.089 (0.915)	0.513		
IOP	0.091 (1.095)	0.181		
Axial length	−0.092 (0.912)	0.675		
Baseline BCVA	0.240 (1.271)	0.764		
Baseline SFT	0.019 (1.020)	**<0.001**	0.009 (1.009)	0.367
Baseline IRLT	0.021 (1.021)	**<0.001**	0.018 (1.018)	**<0.001**
Lens status	0.268 (1.308)	0.556		
Photoreceptor layer disruption	0.478 (1.613)	0.272		
Stage of ERM	0.472 (1.603)	0.080		
Final BCVA	6.310 (55.190)	**0.004**	4.431 (83.987)	**0.039**
Final SFT	0.015 (1.015)	**0.002**	−0.004 (0.996)	0.364
Final IRLT	0.018 (1.018)	**0.003**	0.009 (1.009)	0.438

CI, confidence interval; SE, spherical equivalent; IOP, intraocular pressure; BCVA, best-corrected visual acuity; SFT, subfoveal thickness; IRLT, inner retinal layer thickness. Values in boldface (*p* < 0.050) are statistically significant.
