# Correction: Lee et al. Long-Term Outcome of Epiretinal Membrane Surgery in Patients with Internal Limiting Membrane Dehiscence. *J. Clin. Med.* 2020, *9*, 2470

**DOI:** 10.3390/jcm11154377

**Published:** 2022-07-28

**Authors:** Min-Woo Lee, Il Jung, Yong-Yeon Song, Seung-Kook Baek, Young-Hoon Lee

**Affiliations:** Department of Ophthalmology, Konyang University College of Medicine, Daejeon 35365, Korea; bogus1105@gmail.com (M.-W.L.); jungil@kyuh.ac.kr (I.J.); legend-syy@hanmail.net (Y.-Y.S.); backkka@hanmail.net (S.-K.B.)

The authors would like to correct the keyword ‘bare RNLF’ to ‘RNFL schisis’ in their prior publication [1]. The word ‘bare RNFL’ was first used in a previous study published 2 months before these authors’ paper [2]. Since it is inappropriate to use the keyword without citation, the keyword was replaced as above.

The authors would like to apologize for any inconvenience caused to the readers by these changes.
